# Supplementary material for: Analysis of independent cohorts of outbred CFW mice reveals novel loci for behavioral and physiological traits and identifies factors determining reproducibility
Source: G3 (Bethesda). 2021 Nov 13;12(1):jkab394. doi: 10.1093/g3journal/jkab394 (PMC8728023; doi:10.1093/g3journal/jkab394)
Supplement: jkab394_Supplementary_Figures_Tables [file jkab394_supplementary_figures_tables.pdf]

Table of Contents

Supplemental Figures 2

Supplemental Tables 12

Supplemental Data 16

Supplemental Methods

Genotype Imputation

Reads were aligned to the reference genome assembly (NCBI release 38, mm10). Imputation was carried out using STITCH (Davies et al. 2016) (version = 1.6.0, K = 4, nGen = 100) at 7.07 M bi-allelic autosomal and chromosome X SNPs on the combined set of N = 2073 Oxford + N = 1161 Chicago mice. No samples were removed post-imputation. Genotypes for assessing imputation accuracy were generated on the MegaMUGA array (Collaborative cross consortium 2012) for 48 Oxford and 48 Chicago samples at 77,588 SNPs. After applying standard per-sample (minimum quality scores, removal of samples failing sex checks) and per-SNP (missing less than 5%, Hardy-Weinberg Equilibrium (HWE), p-value greater than $1x10^{-10}$) quality thresholds, genotypes for 44 Oxford and 32 Chicago samples at 73,218 SNPs were available for accuracy assessment. Accuracy was assessed using correlation (r^2) SNP-wise between imputed and true (array) genotypes. Post-imputation SNP QC was carried out for each SNP using each of the combined set of all samples, the Oxford samples only, and the Chicago samples only. Metrics of INFO score and HWE p-value (using hard genotype calls where the maximum posterior genotype probability was greater than 0.9) were calculated for each. Array genotypes were intersected with the imputed genotypes (14,942 SNPs), and only sites with a minimum allele frequency of 1% across each of the three sets kept (13,933 SNPs).

Supplemental Figures

Supplemental Figure 1: Pairwise phenotypic correlations. Phenotypes have the same order on the horizontal and vertical axes. The color of the phenotype label corresponds to whether it is a physiological (blue) or behavioral (red) phenotype. The color corresponds to the Pearson correlation between each pair of phenotypes. Phenotypes within the same category are more correlated to each other than phenotypes in different categories.

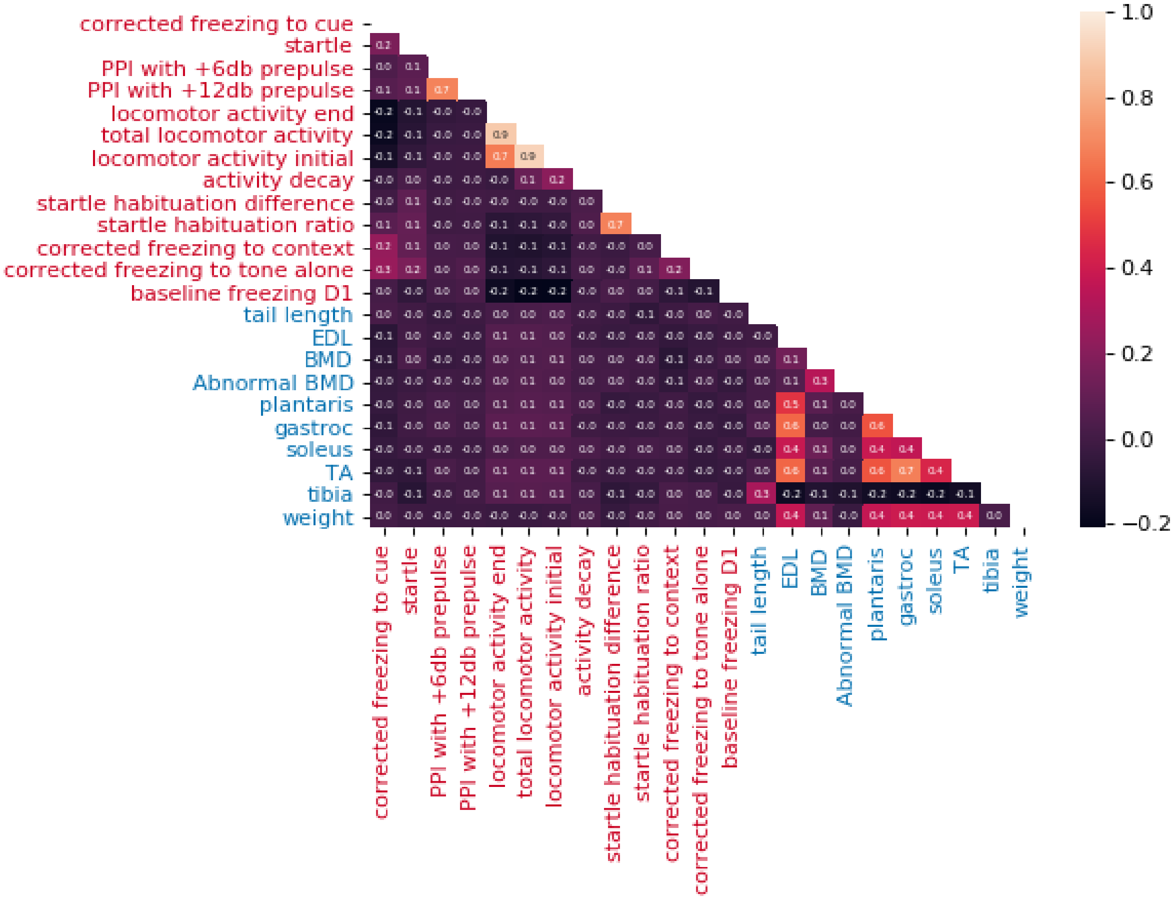

Supplemental Figure 2

Histograms of INFO scores for OX, UC, and mega-analysis are shown in the top row. Windowed accuracy per bin (i.e. only SNPs with that value of the INFO score) are shown in the bottom row.

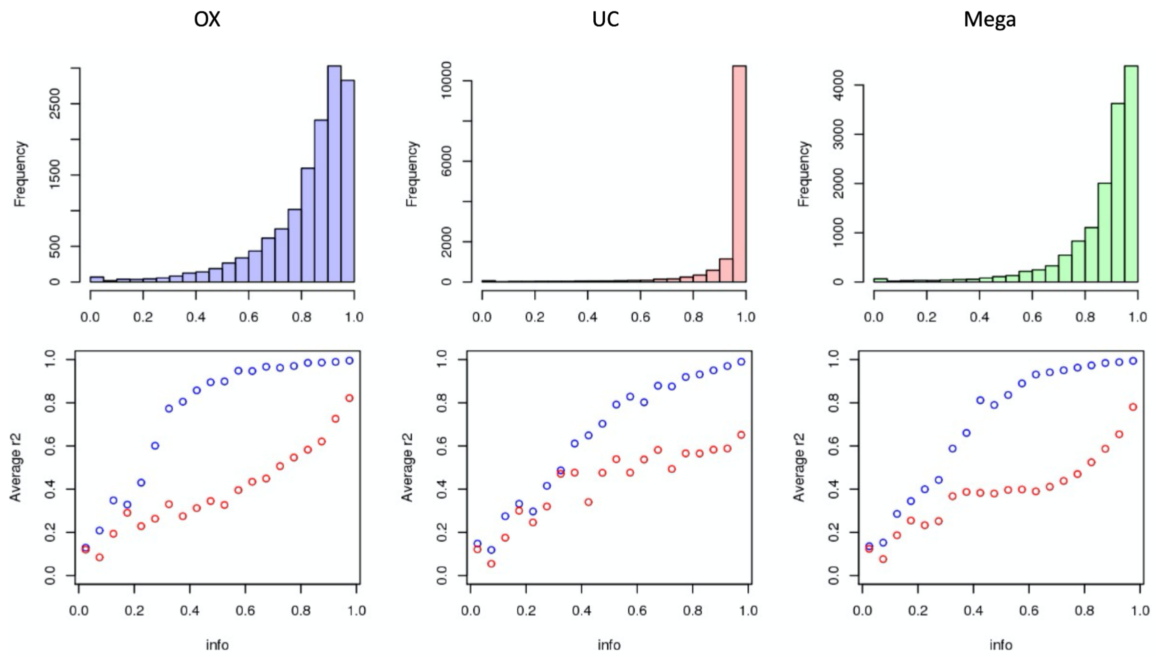

Supplemental Figure 3

Histograms of HWE scores for OX, UC, and mega-analysis are shown in the top row. Windowed accuracy per bin (i.e. only SNPs with that value of the HWE score) are shown in the bottom row.

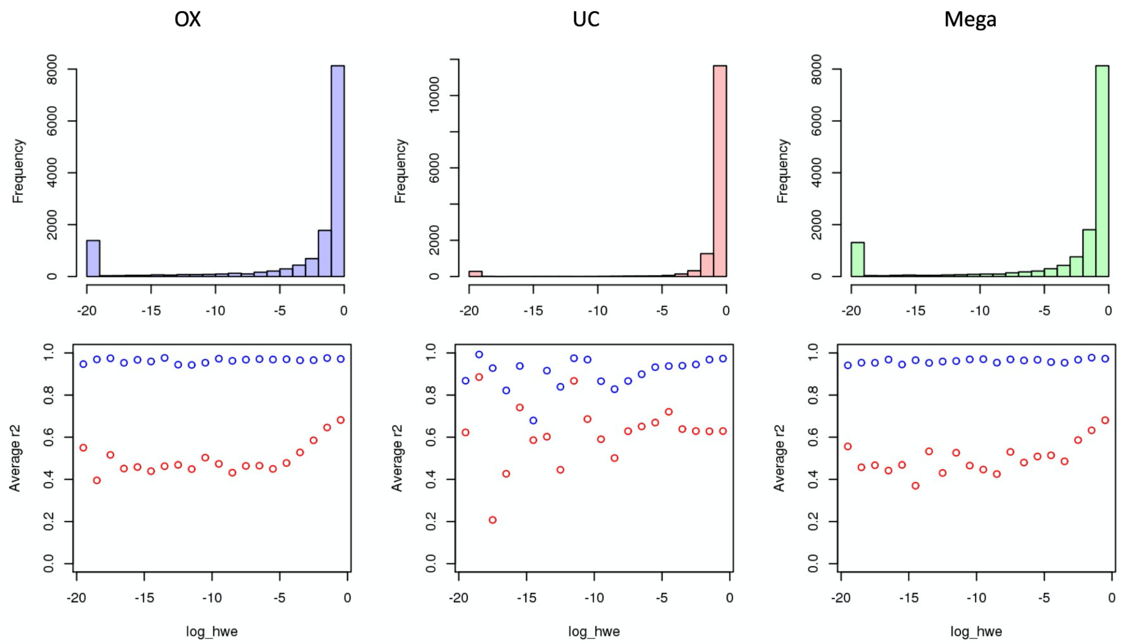

Supplemental Figure 4:

Quantile-quantile (QQ-) plot for total locomotor activity phenotype. The theoretical quantiles of the negative logarithm p-value distribution (horizontal axis) are plotted against the observed distribution (vertical axis). The colors correspond to the mega-analysis (blue), OX (orange), and UC (green) cohorts.

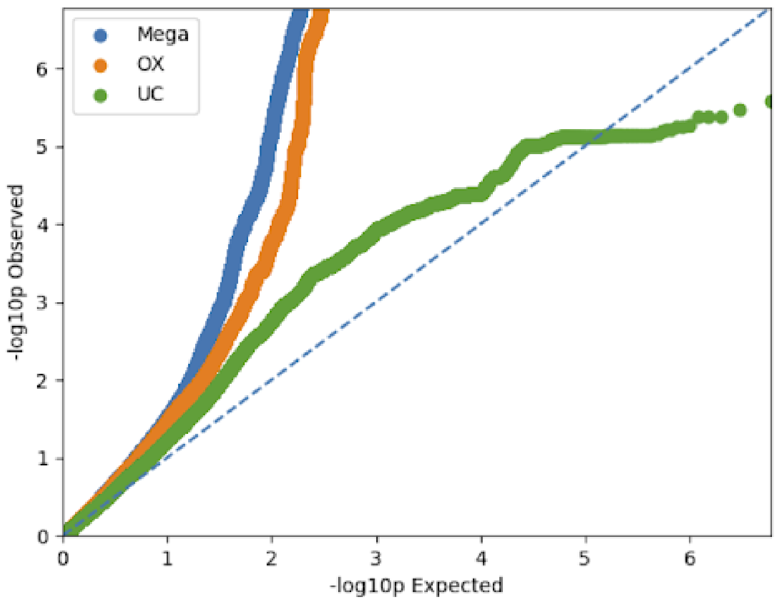
Mega
OX
UC
-log10p Observed
-log10p Expected
0
1
2
3
4
5
6

Supplemental Figure 5: Genetic correlation between all phenotypes of OX1 and OX2 data sets.. Each dot represents the estimated genetic correlation, and the error bars show the 95% confidence intervals obtained using the standard errors.

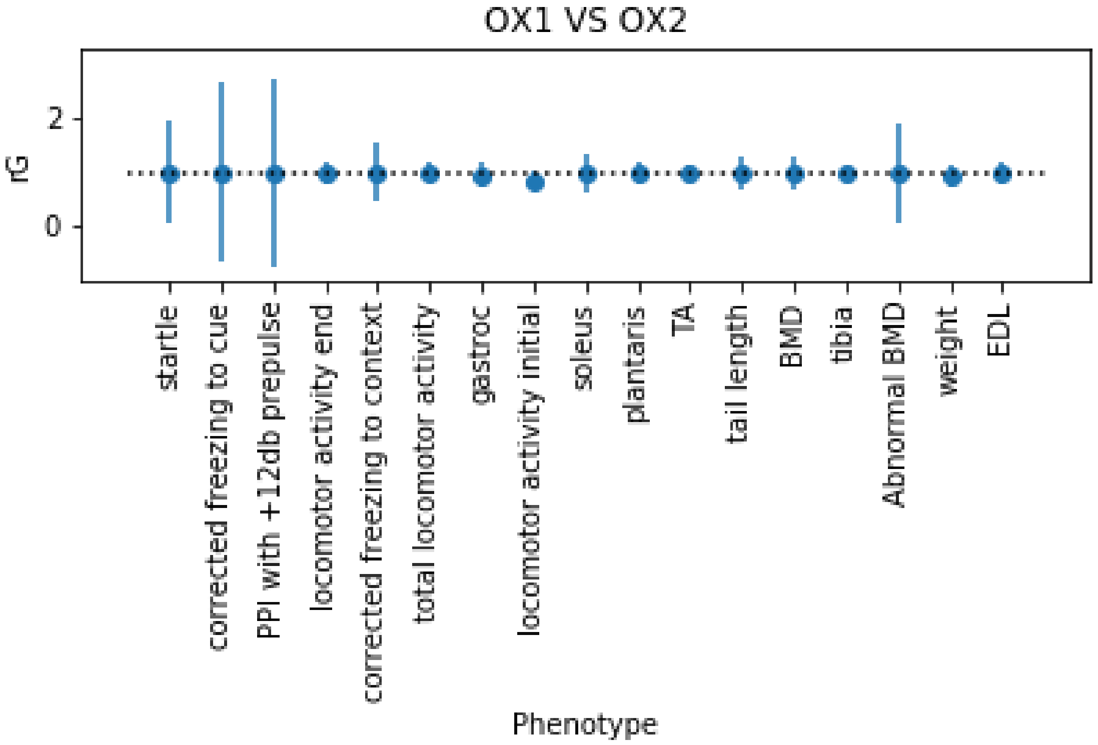

Supplemental Figure 6: Comparison of significance thresholds using decorrelated and naïve methods. We applied permutation-based methods ("decorrelated" and "naïve") to identify significance thresholds in the three cohorts (OX - red, UC - blue, Combined- green). The thresholds obtained using the decorrelated method (solid lines) tend to be more stringent than the thresholds obtained using the naïve method (dotted lines). We use the decorrelated thresholds in our analysis ($\alpha = .05$).

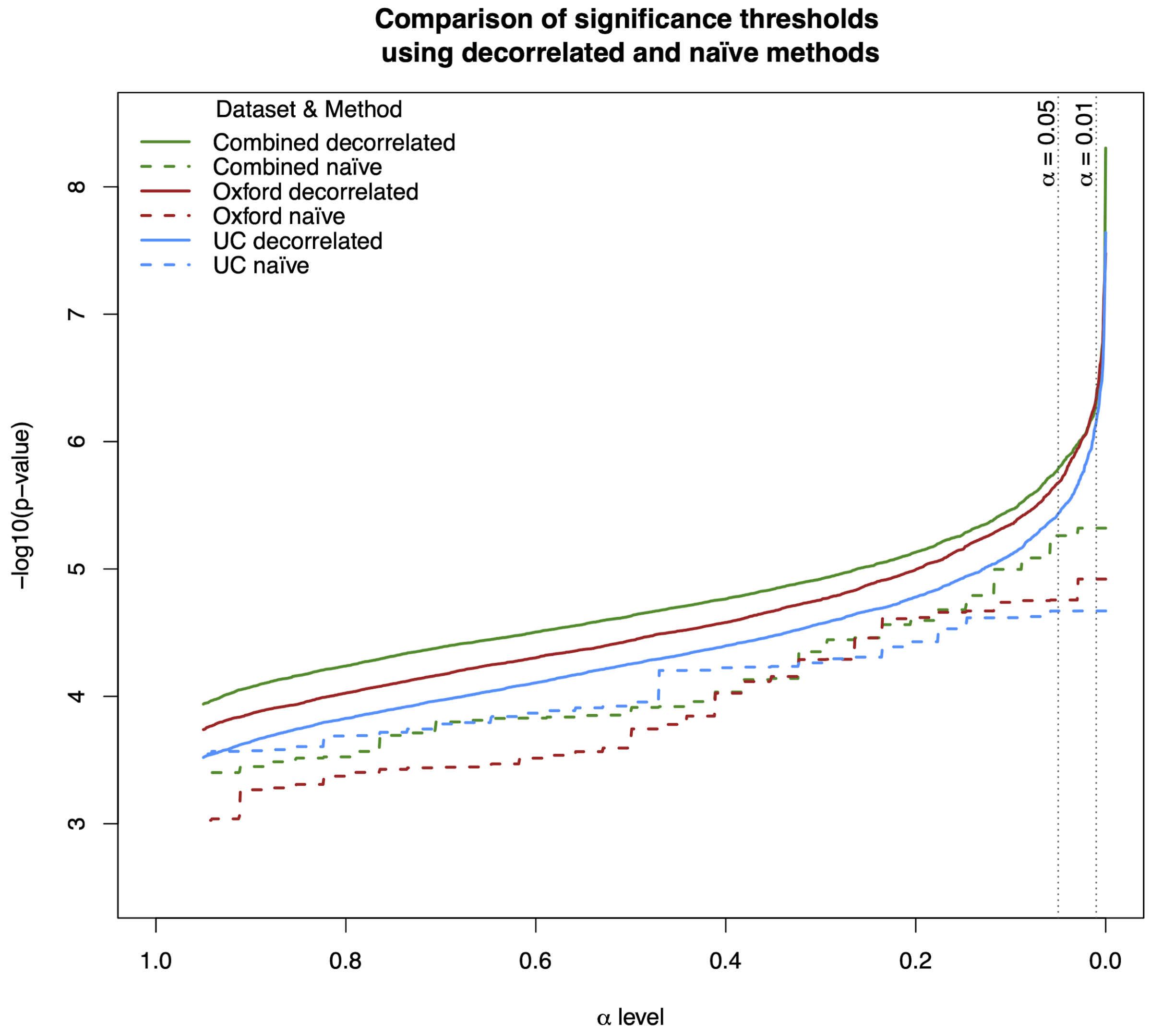

Supplemental Figure 7: Distribution of the widths of the confidence intervals for the mega-analysis QTLs. The median confidence interval size is 1.3 MB.

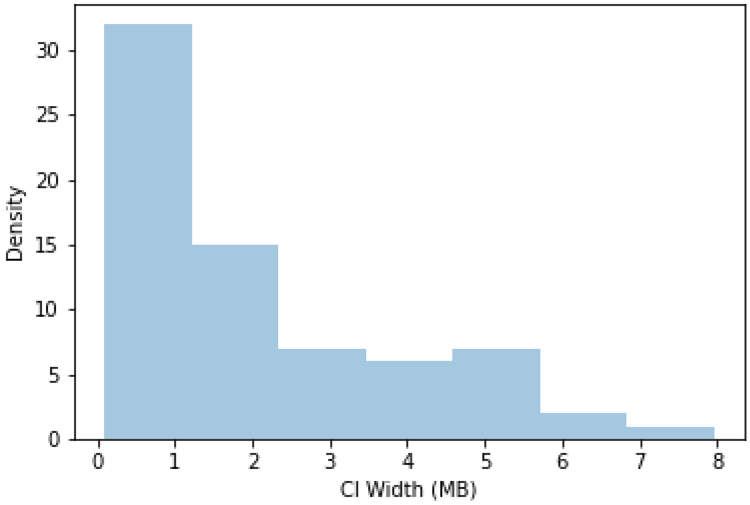

Supplemental Figure 8: We used all variants within the 95% confidence intervals of the lead SNPs as input to the SusieR fine-mapping framework. The number of SNPs used as input to the fine mapping ("Finemapping Input") is on average larger than the number of SNPs output in the credible sets ("Credible Sets"). The median reduction in the number of SNPs from the fine mapping input to the credible sets was 51%.

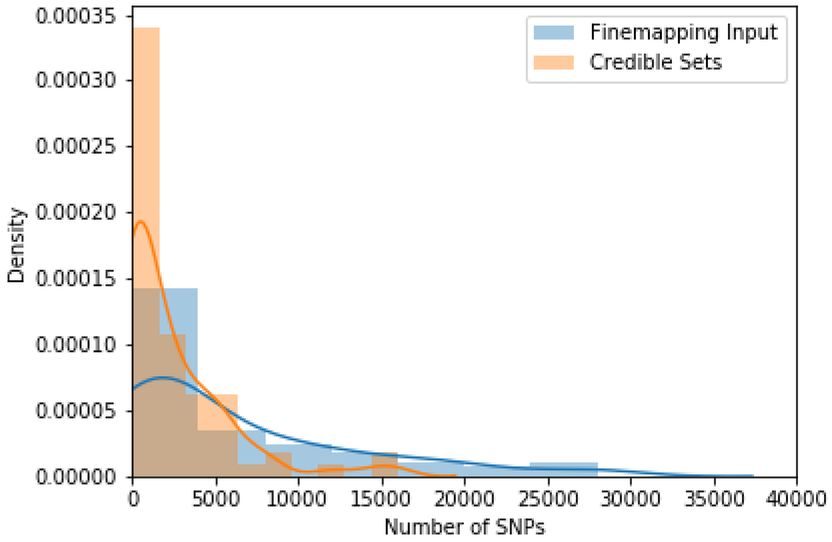

Supplemental Figure 9 : *Co-localization of QTLs for different traits. The horizontal axis lists the QTLs, the vertical axis gives the negative logarithm (base 10) of the association P-value. The color of the text corresponds to the phenotype group of the QTL, with red corresponding to behavior and blue to physiology. The vertical axis shows the negative logarithm p-values of association statistics, where black diamonds correspond to the value of the QTLs. Circles represent the values at other traits, where red corresponds to behavior and blue to physiology. In some cases, behavior QTLs colocalize with physiology QTLs and vice versa. The horizontal black line corresponds to a 5 percent Bonferroni threshold (p< 2.1e-05) that corrects for the number of QTLs and phenotypes tested.*

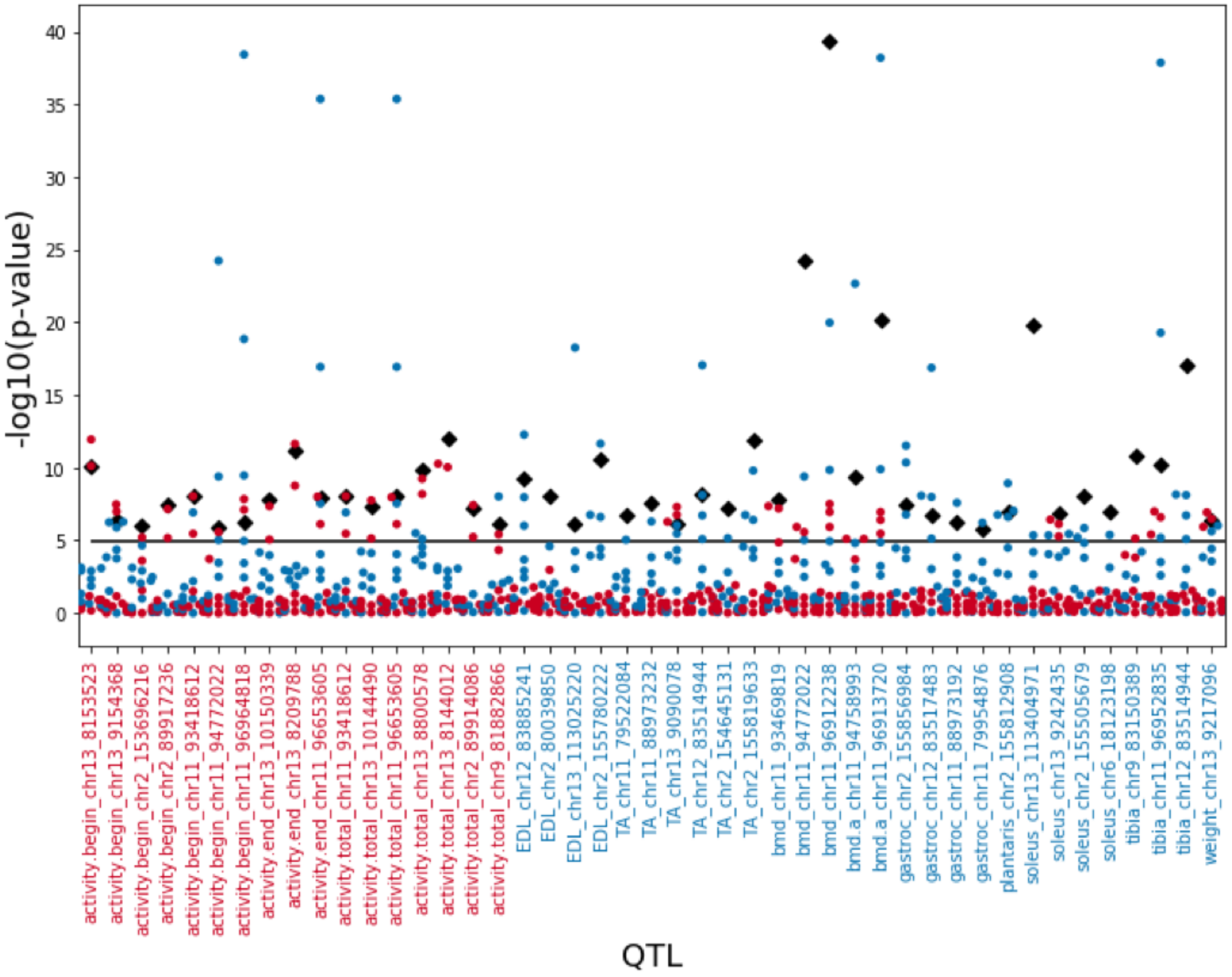

Supplemental Figure 10: Genome-wide representation of QTLs identified for all phenotypes in the two studies, OX and UC. Light grey dots show association for the measures where a QTL was detected. The most significant SNPs at each QTL are marked with a colored dot, where blue represents physiological and red behavioral traits. Some peaks contain multiple QTLs from related traits.

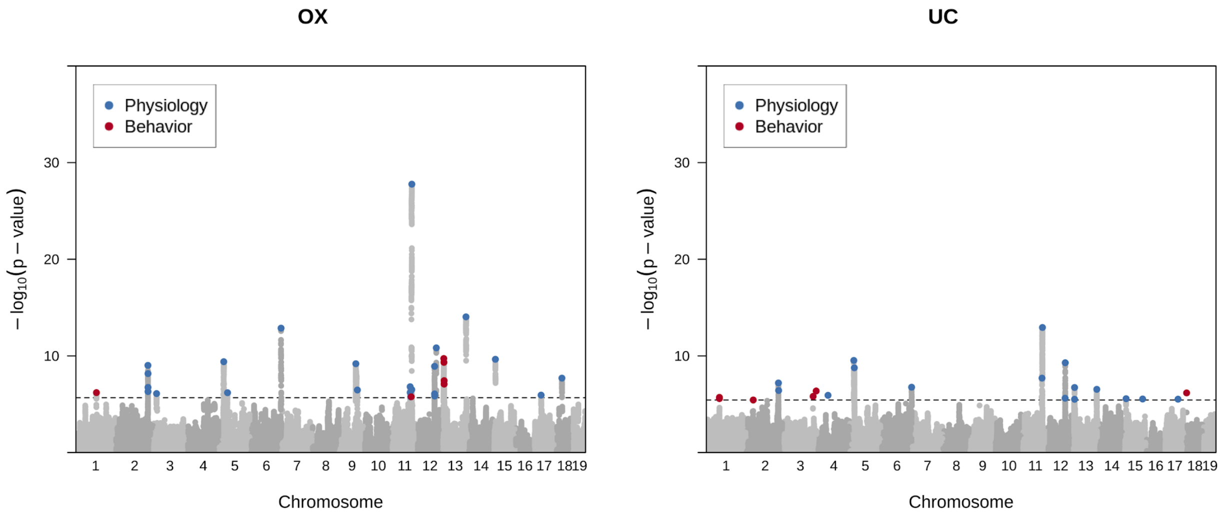

Supplemental Figure 11: We estimated power of each QTL after correcting for Winner's Curse in the OX study, UC study, and mega-analysis ("OX", "UC", and "Mega", respectively). The distributions of the power levels in each study show that the mega-analysis obtained higher power than the component studies.

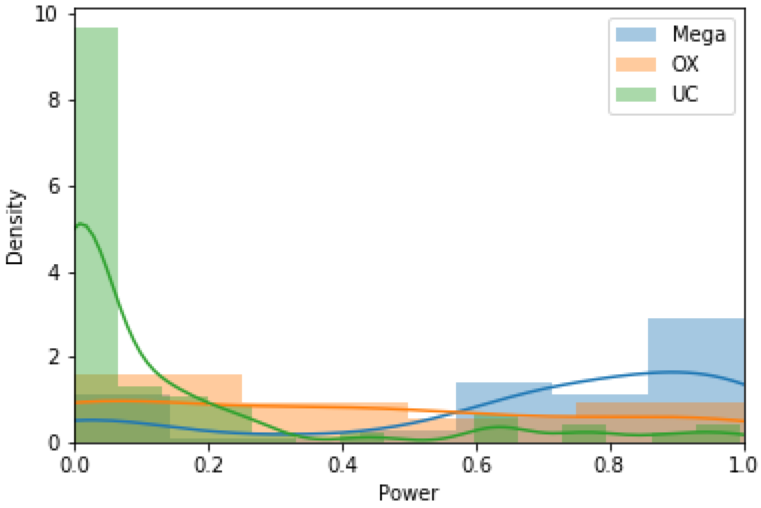

Supplemental Tables

Supplemental Table 1: Phenotype descriptions. We categorized the 23 phenotypes into several categories ("Category"). The sample sizes of the component studies are shown in the "OX" and "UC" columns. The phenotype abbreviations ("id") are used in Supplemental Data Files.

Phenotype name	Category	Description	OX	UC	id
startle habituation ratio	prepulse inhibition	Habituation (calculated as ratio of reduction from first to last startle stimulus)	1682	824	habit.ratio
startle habituation difference	prepulse inhibition	Habituation (calculated as difference first to last startle stimulus	1703	983	habit.diff
startle	prepulse inhibition	Startle	1706	962	startle
PPI with +6db prepulse	prepulse inhibition	PPI with +6db prepulse	1720	936	pp6.ppi
PPI with +12db prepulse	prepulse inhibition	PPI with +12db prepulse	1725	946	pp12.ppi
corrected freezing to context	fear conditioning	% time freezing during context test (corrected for baseline)	1866	1025	fc.context.corr
corrected freezing to tone alone	fear conditioning	% time freezing to tone prior to shock (corrected for baseline)	1873	1035	fc.uncond.freeze.corr
corrected freezing to cue	fear conditioning	% time freezing during cue test (corrected for baseline)	1904	1030	fc.cue.corr
baseline freezing D1	fear conditioning	% time freezing at baseline during training	1874	1035	fc.baseline
activity decay	locomotor activity	Decrease in activity from beginning to end	1899	954	decay.activity
locomotor activity initial	locomotor activity	Activity from 0-15 min	1902	954	activity.begin
total locomotor activity	locomotor activity	Total activity from 0-30 minutes	1900	954	activity.total
locomotor activity end	locomotor activity	Activity from 15-30 minutes	1900	954	activity.end
tail length	physiology	Tail length (cm)	1549	1053	tail.length
TA	physiology	Weight of tibialis anterior (mg)	1828	1057	TA
soleus	physiology	Weight of soleus (mg)	1832	1056	soleus
gastroc	physiology	Weight of gastrocnemius (mg)	1832	1054	gastroc
plantaris	physiology	Weight of plantaris (mg)	1832	1056	plantaris
EDL	physiology	Weight of extensor digitorum longus (mg)	1833	1054	EDL
BMD	physiology	Bone mineral density	1838	960	bmd
tibia	physiology	Length of tibia (mm)	1841	1022	tibia
Abnormal BMD	physiology	Dichotomized bone mineral density	1859	960	bmd.a
weight	physiology	Weight (g)	1865	1038	weight

Supplemental Table 2: Heritability of phenotypes mapped in the mega-analysis. Heritabilities ("heritability") were determined using pruned genetic variants with INFO score > 90. The standard errors of the heritability estimates are shown in column "standard error".

phenotype	h2	h2_se
corrected freezing to cue	0.05	0.02
PPI with +12db prepulse	0.05	0.02
corrected freezing to context	0.06	0.02
Abnormal BMD	0.06	0.02
startle	0.08	0.02
gastroc	0.15	0.02
locomotor activity end	0.15	0.02
tail length	0.15	0.02
locomotor activity initial	0.17	0.02
BMD	0.18	0.02
total locomotor activity	0.18	0.02
plantaris	0.18	0.02
weight	0.19	0.02
soleus	0.19	0.02
EDL	0.21	0.02
TA	0.22	0.02
tibia	0.33	0.03

Supplemental Table 3: 95% confidence intervals of QTLs identified in mega-analysis. For each QTL in the mega-analysis, we compiled the estimated effect size in the mega-analysis ("beta"), the standard error on the effect size ("se"), and the negative logarithm of the p-value ("logP"). We also computed 95% confidence intervals on each QTL. The starting position of the

confidence intervals are in the "from.bp" column, and the ending position of the confidence intervals are in the "to.bp" column. The coordinates are 1-indexed and inclusive. These coordinates include all variants in perfect LD with the simulation based confidence intervals.

Supplemental Table 4: Nonsynonymous mutations identified through fine-mapping of mega-analysis QTLs. We identified variants implicated by the SusieR fine-mapping method that were nonsynonymous mutations in genes. These genes are likely involved in the tested traits and in many cases have been independently identified in similar GWAS studies or mouse knockout studies ("GWAS Phenotype" and "KO Mouse Phenotype"). The references for these previously published studies are provided in the "References" column. The lead QTLs are shown in the "QTLs" column, and the confidence levels for the QTLs are shown in the "confidence column". 1 corresponds to the highest level of confidence, and 4 corresponds to the lowest level of confidence. The confidence levels were obtained using a combination of replication data, estimated level of confounding, and power in the mega-analysis.

Supplemental Table 5: Colocalization of mega-analysis QTLs. For each QTL in the mega-analysis, we computed the effect size, standard error on the effect size, and log p-value for every other trait (columns "beta", "se", and "logP", respectively). The phenotype in which the QTL was found in the mega-analysis is in the "phenotype_qtl" column, and the other phenotypes that colocalize are in the "phenotype" column. We used a Bonferroni corrected threshold of 0.05 to identify QTLs that significantly colocalized with multiple traits.

Supplemental Table 6: Confidence levels of QTLs identified in mega-analysis. For each QTL in the mega-analysis, we compiled the estimated effect size in the mega-analysis ("combined.beta") and the standard error ("combined.se"). We also computed the negative

logarithm of the p-values for the mega-analysis and component studies ("combined.logp", "ox.logp", "uc.logp"). We corrected the bias due to Winner's Curse and used the corrected effect size estimates to compute the power of the association in each study ("combined.power", "ox.power", "uc.power").

Supplemental Table 7: Summary of QTLs in each confidence category ("Category"). The total number of QTLs identified is in the "QTLs" column, and the number of phenotypes is shown in "Phenotypes." We identified a number of variants that were predicted to be causal from our fine-mapping analysis. We report the total number of variants that were nonsynonymous mutations ("Nonsyn"), and the number of genes affected in ("Genes").

Supplemental Data

Supplemental Data 1: Phenotypes

Supplemental Data 2: Genotypes

Supplemental Data 3: Genome-wide Association Studies

Supplemental Data 4: Fine-mapping (lists of SNPs in causal set for each QTL)
